# Pit cells exclusively kill P815 tumor cells by the perforin/granzyme pathway

**DOI:** 10.1186/1476-5926-2-S1-S58

**Published:** 2004-01-14

**Authors:** David Vermijlen, Dianzhong Luo, Christopher J Froelich, Jan P Medema, Jean A Kummer, Erik Willems, Filip Braet, Eddie Wisse

**Affiliations:** 1 Laboratory for Cell Biology and Histology, Vrije Universiteit Brussel (VUB), Laarbeeklaan 103, 1090 Brussels, Belgium; 2 Evanston Northwestern Healthcare Research Institute, Evanston, Illinois, USA; 3 Department of Immunohematology and Bloodtransfusion, Leiden University Medical Center, Leiden, The Netherlands; 4 Institute for Biochemistry, BIL Biomedical Research Center, University of Lausanne, Epilanges, Switzerland; 5 Present address: Department for Molecular Biomedical Research, Molecular Cell Biology Unit, Ghent University (UGhent), Technologiepark 927, 9052 Zwijnaarde, Belgium

## Introduction

Hepatic natural killer (NK) cells, also known as pit cells, are located in the liver sinusoids, adhering to the endothelial cells (LSECs), and are thus in a strategic position to kill arriving metastasizing tumor cells [1-3]. NK cells of different tissue origin (blood, spleen, liver) appear to have different levels of cytotoxicity. Lower levels can be enhanced by lymphokines such as interleukin-2 (IL-2) or IL-12, providing lymphokine-activated killer (LAK) cells [1]. P815 mastocytoma cells were found to be resistant to the induction of cytolysis (quantified by ^51^Cr release) by NK cells from spleen or blood, but are sensitive to hepatic NK and LAK cells [[1,3] and references therein]. Hepatic NK cells therefore might be considered as naturally activated LAK cells.

Cytotoxic lymphocytes (NK cells, LAK cells, cytotoxic T cells, NK-T cells) use the FasL and the perforin/granzyme pathway to kill target cells [3]. FasL on effector cells binds Fas present on the target cell membrane, which results in oligomerization of Fas and activation of caspase 8. Perforin and granzymes, of which granzyme B is the most potent, reside in granules of the cytotoxic lymphocytes and are released by exocytosis. Intracellular delivery of granzyme B results in the initiation of the caspase cascade by proteolytic activation of caspase 3, either directly [4] or through a mitochondrium-dependent pathway [5]. Caspases play a central role in the execution of apoptosis [4]. In this study, we investigated the mechanism hepatic NK cells use to kill P815 cells.

## Methods

**P815**, a mouse mastocytoma cell line, was maintained in culture medium consisting of DMEM (42430, GIBCO, Life Technologies, Belgium) supplemented with 10 % fetal bovine serum (Eurobiochem, Bierges, Belgium), sodium pyruvate (1 mmol/L), penicillin (100 U/ml), streptomycin (100 U/ml), and L-glutamine (0.2 mmol/L) (GIBCO, Life Technologies).

**Hepatic NK cells **were isolated from male Wistar rats (Proefdierencentrum, K.U.L., Leuven, Belgium) of 12–16 weeks old weighing ca. 300 g, as described previously [6,7].

**Transmission electron microscopy (TEM) **was performed as described [8].

**Quantitative DNA fragmentation assay **was performed as described at an E/T ratio of 10/1 and 3 h co-incubation [8].

### ^51^Cr release assay

Cytolysis was measured in a 4 h ^51^Cr release assay as described previously [9]. DCI (3,4-dichloroisocoumarin) and EGTA were purchased from ICN (Asse-Relegem, Belgium) and Z-VAD-FMK (Z-Val-Ala-Asp(OMe)-fluoromethylketone) from Bachem (Bubendorf, Switzerland).

## Results and Discussion

**Figure 1:**
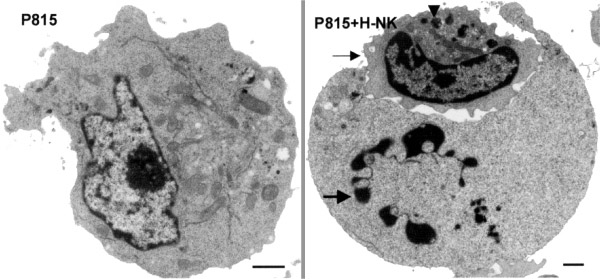
Hepatic NK cells induce apoptosis in P815 cells as shown by TEM. P815, P815 cells in medium only; P815+H-NK, P815 cells coincubated for 3 h with hepatic NK cells at an E/T ratio of 10/1. When P815 cells are coincubated with hepatic NK cells (thin arrow), nuclei of P815 cells become condensed and fragmented (thick arrow). Note the granules present in the hepatic NK cells (arrowhead). Bar: 1 –m. (From reference 8, with permission.)

Hepatic NK cells induced nucleus condensation and fragmentation in P815 cells, as shown by fluorescent nuclear staining (data not shown) and TEM (Fig. 1). Chromatin was condensed into masses that abutted the inner surface of the nuclear envelope and was accompanied by nuclear fragmentation (Fig. 1). Using DNA fragmentation and ^51^Cr release we verified that hepatic and not splenic NK cells kill P815 cells [8].

**Figure 2:**
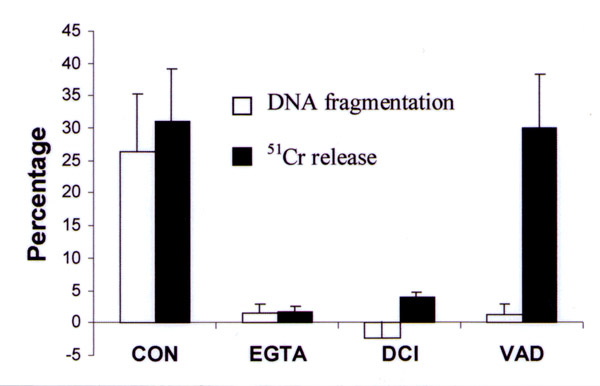
The effect of inhibitory compounds on the killing of P815 cells by hepatic NK cells as determined by DNA fragmentation and ^51^Cr release. CON, control; EGTA, 5 mmol/L EGTA present during coincubation; DCI, preincubation of hepatic NK cells with 50 micromolar/L DCI for 30 minutes, no DCI present during coincubation; VAD, preincubation of P815 cells with 160 micromolar/L Z-VAD-FMK for 30 minutes, 80 micromolar /L present during coincubation. Values are means of three independent experiments. Error bars, SD. (From reference 8, with permission.)

We showed that P815 cells are sensitive to both the FasL and perforin/granzyme pathway and hepatic NK cells express FasL, perforin and granzyme B [8]. Several approaches, distinguishing the FasL and perforin/granzyme pathway, were used to determine how hepatic NK cells induce apoptosis in P815 targets. Chelation of extracellular Ca^2+ ^with EGTA (5 mmol/L), a treatment known to block granule exocytosis and the action of perforin [10], completely abolished DNA fragmentation and ^51^Cr release (Fig. 2). Preincubation of the effector cells with DCI (50 micromolar for 30 minutes), an inhibitor of granzymes in intact cells [11,12], completely inhibited DNA fragmentation and substantially blocked ^51^Cr release (Fig. 2). Consistent with previous reports [13,14], the general caspase inhibitor Z-VAD-FMK abrogated DNA fragmentation but ^51^Cr release was unaffected (Fig. 2). These results clearly demonstrate that P815 cells are exclusively killed by the granule pathway, whereas other cytotoxic lymphocytes can use both the FasL and perforin/granzyme pathway to kill this target [15-17].

**Figure 3:**
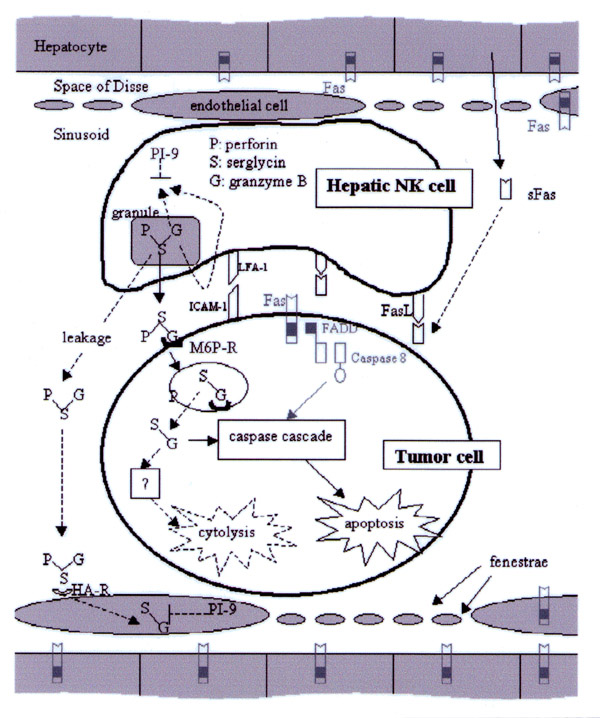
Model of hepatic NK cell-mediated tumor cell killing. For explanation: see text. The blocked FasL pathway is indicated in gray. Dashed lines indicate hypothetical relations. FADD, Fas-associated death domain factor; FasL, Fas ligand; LFA-1, leukocyte function associated molecule-1; G, granzyme B; HA-R, hyaluronan receptor; ICAM-1, intercellular adhesion molecule-1; M6P-R, mannose 6 phosphate receptor (cation-independent); P, perforin; PI-9, protease inhibitor 9; S, serglycin; sFas, soluble Fas. (From reference 8, with permission.)

We showed that hepatic NK cells and LSECs, which are in contact with the hepatic NK cells, are strongly positive for the granzyme B inhibitor, serine protease inhibitor PI-9/SPI-6, and that expression of this inhibitor in target cells results in complete resistance to hepatic NK cell-induced apoptosis [8]. Based on these results, we put forward following model for hepatic NK cell-mediated killing (Figure 3): When a tumor cell enters a liver sinusoid, it is mechanically trapped and/or adheres to LSECs. Hepatic NK cells adhere to tumor cells by adhesion molecules like LFA-1. sFas produced by hepatocytes blocks FasL on the hepatic NK cells, preventing possible harmful effects on the FasL-sensitive LSECs and hepatocytes. On the other hand, highly expressed perforin and granzyme B, as a complex with serglycin as a scaffold [18], are released by granule exocytosis in the space formed between the NK-tumor conjugate. Damaging of other cells (e.g. hepatocytes) caused by leakage of granzyme B/perforin is prevented by the very efficient endocytic uptake of the granzyme B/serglycin/perforin complex by the HA-R expressed on LSECs [19]. LSECs are protected from the action of granzyme B by strong expression of the granzyme B inhibitor PI-9/SPI-6 [8]. Granzyme B, presumably taken up by the M6P-R [20], induces apoptosis in the tumor cell by activating the caspase cascade. On the other hand, cytolysis (^51^Cr release) is induced by a caspase-independent mechanism.
